# Larvicidal activity of *Zanthoxylum acanthopodium* essential oil against the malaria mosquitoes, *Anopheles anthropophagus* and *Anopheles sinensis*

**DOI:** 10.1186/s12936-018-2341-2

**Published:** 2018-05-15

**Authors:** Qi He, Wenxia Wang, Liang Zhu

**Affiliations:** College of Light Industry and Food, South China University of Technology, Guangzhou, Guangdong Province China

**Keywords:** *Zanthoxylum acanthopodium*, Essential oil, *Anopheles anthropophagus*, *Anopheles sinensis*, Larvicidal activity

## Abstract

**Background:**

*Zanthoxylum acanthopodium* has insecticidal effect in Chinese traditional medicine. In this study, the essential oil from the dried *Zanthoxylum* plant was used as a larvicidal compound against the malaria mosquitoes, *Anopheles anthropophagus* and *Anopheles sinensis.*

**Methods:**

Compounds in the *Zanthoxylum* essential oil were investigated by gas chromatography and mass spectroscopy (GC–MS). The larvicidal bioassays of the whole oil, as well as the main compounds in the oil (estragole and eucalyptol) were performed using WHO method.

**Results:**

In total, 63 main compounds (99.32%) were found in the oils, including estragole (15.46%), eucalyptol (10.94%), β-caryophyllene (5.52%), *cis*-linalool oxide (3.76%), *cis*-limonene oxide (3.06%). A dose-dependent effect on mortality was recorded with increasing concentrations of essential oil and compounds increasing mortality of the larvae. Larvicidal bioassays revealed that 24 h LC_50_ of the whole essential oil was 36.00 mg/L and LC_90_ was 101.49 mg/L against *An. anthropophagus*, while LC_50_ was 49.02 mg/L and LC_90_ was 125.18 mg/L against *An. sinensis*. Additionally, 24 h LC_50_ of estragole were 38.56 and 41.67 mg/L against *An. anthropophagus* and *An. sinensis*, respectively, while the related LC_90_ were 95.90 and 107.89 mg/L. LC_50_ of eucalyptol were 42.41 and 45.49 mg/L against *An. anthropophagus* and *An. sinensis*, while the related LC_90_ were 114.45 and 124.95 mg/L.

**Conclusion:**

The essential oil of *Z. acanthopodium* and its several major compounds may have potential for use in the control of malaria mosquitoes.

## Background

Malaria, a widespread disease in tropical and subtropical regions (including much of sub-Saharan Africa, Asia, and the Americas), can cause symptoms that include fever, headache, vomiting, and even progress to coma or death [1]. As the World Health Organization (WHO) estimated, there were 216 million documented cases and more than 445,000 deaths from malaria in 2016 [2]. As a mosquito-borne infectious disease, malaria can be caused by a bite from an infected mosquito, which can introduce the malaria parasites into the circulatory system of the human and ultimately to the liver, where they mature and reproduce [3].

Among mosquitoes, *Anopheles* is the only vector of human malaria [4] and, of these, 30–40 species commonly transmit parasites of the genus *Plasmodium* [5]. *Anopheles anthropophagus* and *Anopheles sinensis* have been chosen as the test target mosquito species in this research, because they are rife and harmful in China. The mosquito *An. sinensis* is a member of the *Anopheles hyrcanus* group, distinguished from other series by the presence of pale bands (usually four) on the palpi and a tuft of dark scales on the clypeus on each side. *Anopheles anthropophagus* is a major vector in central China, able to spread malaria 18–20 times better than *An. sinensis* [6, 7]. The larvae of *An. anthropophagus* and *An. sinensis* live on the surface of water. Compared to adults, the ability of larvae to escape the insecticide is limited, so the time from larvae to adult is an important period to control mosquitoes [8].

Generally, mosquito larval control is carried out by conventional synthetic insecticides, which have high efficiency and low cost. Their efficacy may decay gradually because mosquitoes develop resistance against insecticides [9]. By contrast, plant essential oils, often obtained from the plant by steam distillation or hydrodistillation, constitute a rich source of bioactive compounds [10], and have emerged as a good alternative larvicidal or adulticidal agents with fewer insect resistance problems [11]. Biodegradable and nontoxic, they can be used as an eco-friendly tool as well as an economical agent in vector control [12].

*Zanthoxylum acanthopodium* is a shrub or small tree in the *Zanthoxylum* genus of *Citrus* family, native to southern China, Bangladesh, Bhutan, northern India, Nepal, Laos, Burma, northern Thailand Vietnam, Indonesia, and Peninsular Malaysia. It has 5–13 alternate leaves with serrated edge (3–8 cm long and 1–2 cm wide) and axillary inflorescences with densely globose clusters of unisexual flowers [13]. Much like the closely related *Zanthoxylum piperitum*, it has similar tongue-numbing characteristic seed pericarps, which are often used as spices in cooking, and traditional Chinese medicine reported great effect on contraception, relieving pain and killing parasites [13]. Additionally, the hydrodistilled essential oil from *Zanthoxylum acanthopodium*, with many active ingredients, is a novel agent with special effect. In the present study, the chemical composition of the essential oil from *Zanthoxylum acanthopodium* was determined and its larvicidal activity was evaluated against *An. anthropophagus* and *An. sinensis*.

## Methods

### Plant material

The aerial parts of *Zanthoxylum acanthopodium* were collected from 33 families of plants in the Ailao Mountains, Yunnan Province, China, in May 2013. Plant specimens were identified by Dr. Yin-zhang Zhou. The plants were dried under shade (at room temperature). The voucher specimen (No. IBSC/0387990) was deposited in the South China Botanical Garden, Chinese Academy of Sciences.

### Isolation of the essential oil

The air-dried plant materials (2000*g*) of *Zanthoxylum acanthopodium* were chopped and subjected to hydrodistillation for 6 h using a Clevenger type apparatus. The obtained oils were dried over hydrous sodium sulfate for 24 h, filtered, and then stored at 4 °C in brown sealed glass vials until tested.

### GC analysis

An Agilent HP-6890 gas chromatograph (Agilent Technologies, Palo Alto, CA, USA) with a HP-5 5% phenylmethylsiloxane capillary column (30 m × 0.25 mm i.d., 0.25 μm film thickness) and an FID detector was used for GC–FID analysis. Helium gas (1 mL/min) was used as the carrier gas. Injector and mass transfer line temperatures were set at 250 and 280 °C, respectively. The EO solution (1 μL) in hexane was injected and analysed under the following column conditions: initial column temperature of 40 °C for 1 min, increased to 250 °C at a 3 °C/min, and maintained at 250 °C for 20 min.

### GC–MS analysis

The oils were quantitatively and qualitatively analysed using a GC–MS 6890-5975 system (Agilent Technologies, Palo Alto, CA, USA) equipped with a HP-5 MS fused silica capillary column (30 m × 0.25 mm i.d., 0.25 μm film thickness). For GC–MS detection, an electron ionization system with ionization energy of 70 eV was used. Helium gas (1 mL/min) was used as carrier gas. Injector and mass transfer line temperatures were set at 250 and 280 °C, respectively. EO solution (1 μL) in hexane was injected and analysed under the following column conditions: initial column temperature at 40 °C for 1 min, increased to 250 °C at a 3 °C/min heating ramp, and maintained at 250 °C for 20 min. Kovats indices were calculated for all of the volatile components by using a homologous series of *n*-alkanes (C_8_–C_25_) in an HP-5 MS column. The major oil components were identified by co-injection with standards (whenever possible) and confirmed using the Wiley (V.7.0) and National Institute of Standards and Technology V.2.0 GC–MS library on the basis of Kovats indices. The relative concentration of each compound in the oil was quantified on the basis of the peak area integrated in the analysis program.

### Mosquito larvicidal assay

The eggs of *An. sinensis* and *An. anthropophagus* were received from Centre for Disease Control and Prevention of Guangdong Province, China. Twenty-five larvae were reared in 250 mL glass beaker and fed with Brewer’s yeast/dog biscuit (1:3) [14, 15]. The beaker with larvae were maintained at 27 ± 2 °C, 75 ± 2% relative humidity and photoperiod of 14:10 (L:D) h. Larvicidal activity of the essential oil and of the main compounds (estragole and eucalyptol) isolated from the essential oil of *Zanthoxylum acanthopodium* were evaluated according to the standard procedures suggested by the WHO [16]. The essential oil and compounds were dissolved in 1 mL of DMSO solution and prepared into different concentrations (25, 50, 75, 100 125, and 150 ppm) using distilled water. For comparison commercial larvicide Pylarvex (Pyrethrum Board of Kenya) was used as a positive control. Twenty larvae of early fourth-instar stage were used in the larvicidal assay and five replicates were maintained for each concentration. During this experiment no food was offered to the larvae. The larval mortality was calculated after the 24 h exposure period.

### Statistical analysis

The average larval mortality data were subjected to probit analysis for calculating LC_50_, LC_90_ and other statistics at 95% fiducial limits of upper confidence limit and lower confidence limit, and Chi square values were calculated by using the software using Statistical Package of Social Sciences (SPSS) 13.0 for windows, the significance level was set at P < 0.05.

## Results and discussion

### Chemical composition of the essential oil

The steam distillation of 2000*g* of dried plant material yielded 7.2 mL (0.36%, v/w) of a light- yellow oil with a distinct smell. The GC–MS analysis of *Zanthoxylum acanthopodium* essential oil revealed 63 components representing 99.32% of the oil (Table 1). The composition was 13.98% monoterpene hydrocarbon fraction, 24.24% oxygenated monoterpene fraction, 16.60% sesquiterpene hydrocarbon fraction, 13.75% oxygenated sesquiterpenoid fraction, 21.57% phenylpropanoids and 9.18% others. The main components of the oil were estragole (15.46%), eucalyptol (10.94%), β-caryophyllene (5.52%), *cis*-linalool oxide (3.76%), *cis*-limonene oxide (3.06%).

**Table 1:** Chemical composition of essential oil of *Zanthoxylum acanthopodium*

Peak no.	RI^a^	Components	%RA^b^	Identification methods
		Monoterpene hydrocarbons	13.98	
1	930	α-Thujene	0.62	MS, RI
2	936	α-Pinene	1.28	MS, RI
3	973	Sabinene	2.63	MS, RI
4	986	β-Myrcene	0.41	MS, RI
5	1012	3-Carene	2.76	MS, RI
6	1024	β-Phellandrene	1.36	MS, RI
7	1030	Limonene	2.47	MS, RI
8	1040	(Z)-β-Ocimene	1.22	MS, RI
9	1059	γ-Terpinene	1.23	MS, RI
		Oxygenated monoterpenes	24.24	
10	1036	Eucalyptol	10.94	MS, RI, Co
11	1078	*cis*-Linalool oxide	3.76	MS, RI
12	1099	Linalool	5.10	MS, RI, Co
13	1120	*cis*-4-Isopropyl-1-methyl-2-cyclohexen-1-ol	0.25	MS, RI
14	1143	Camphor C10H16O	1.91	MS, RI
15	1196	Citronellol	0.60	MS, RI
16	1255	Piperitone	0.28	MS, RI
17	1257	Geraniol	0.73	MS, RI
18	1270	Perillaaldehyde	0.67	MS, RI
		Sesquiterpene hydrocarbons	16.60	
19	1335	δ-Elemene	0.79	MS, RI
20	1351	a-Cubebene	0.28	MS, RI
21	1375	Isoledene	0.46	MS, RI
22	1384	β-Bourbonene	0.92	MS, RI
23	1388	β-Cubebene	0.63	MS, RI
24	1390	β-Elemene	0.47	MS, RI
25	1418	β-Caryophyllene	5.52	MS, RI, Co
26	1431	2-Norpinene	0.66	MS, RI
27	1438	E-α-Bergamotene	0.67	MS, RI
28	1458	Seychellene	0.28	MS, RI
29	1458	(E)-b-Farnesene	0.38	MS, RI
30	1474	γ-Muurolene	0.40	MS, RI
31	1486	Germacrene D	2.81	MS, RI
32	1493	Zingiberenol	0.63	MS, RI
33	1498	Bicyclogermacrene	0.34	MS, RI
34	1514	trans-γ-cadinene	0.73	MS, RI
35	1525	β -Sesquiphellandrene	0.63	MS, RI
		Oxygenated sesquiterpenes	13.75	
36	1130	*cis*-Limonene oxide	3.06	MS, RI
37	1549	Elemol	1.65	MS, RI
38	1562	(trans)-Nerolidol	0.19	MS, RI
39	1575	Spathulenol	1.33	MS, RI
40	1578	Caryophyllene oxide	2.27	MS, RI
41	1587	Viridiflorol	0.83	MS, RI
42	1606	α-Humulene epoxide II	0.75	MS, RI, Co
43	1639	Isospathulenol	0.56	MS, RI, Co
44	1648	β-Eudesmol	2.46	MS, RI
45	1654	α-Eudesmol	0.27	MS, RI
46	1666	Bulnesol	0.38	MS, RI
		Phenylpropanoids	21.57	
47	1045	Benzene acetaldehyde	0.85	MS, RI
48	1229	Estragole	15.46	MS, RI, Co
49	1286	Bornyl ester	0.7	MS, RI
50	1308	Carvacrol	0.68	MS, RI
51	1315	2-Methoxy-4-vinylphenol	0.32	MS, RI
52	1357	Eugenol	1.32	MS, RI
53	1408	Methyl eugenol	0.61	MS, RI
54	1534	α-Calacorene	0.38	MS, RI
55	1620	Dill apiole	1.25	MS, RI
		Others	9.18	
56	852	*trans*-2-Hexenal	2.73	MS, RI
57	982	1-Octen-3-ol	1.24	MS, RI
58	1062	trans-2-Octenal	0.48	MS, RI
59	1368	α-Terpinyl acetate	0.57	MS, RI
60	1385	Geranyl acetate	1.34	MS, RI
61	1396	*cis*-Jasmone	0.85	MS, RI
62	1702	2-Hexadecanol	1.34	MS, RI
63	1971	*n*-Hexadecanoic acid	0.63	MS, RI
		Total identified (%)	99.32	

*RI* retention index, *MS* mass spectrum, *Co* co-injection with authentic compound

^a^Retention index relative to n-alkanes on HP-5 MS capillary column

^b^Relative area (peak area relative to the total peak area)

In the *Zanthoxylum acanthopodium* essential oil, main compounds were estragole, eucalyptol and β-caryophyllene. They were often reported in the essential oil extracted from different kinds of *Zanthoxylum* plants. Estragole is a component isolated from various trees and plants and its extraction from *Zanthoxylum* plants has been described but is relatively rare. Wang et al. [17] showed that in the essential oil of fresh fruits of *Zanthoxylum schinifolium*, estragole was the major compound (69.52%). Eucalyptol was common in many plants including *Zanthoxylum* plants in related literature. Liu et al. [18] showed that the essential oil of *Zanthoxylum avicennae* leaves and stems had high content of monoterpenoids including 53.05% eucalyptol. Prieto et al. [19] determined the major constituents of *Zanthoxylum monophyllum* oil were eucalyptol (9.19%); Eiter et al. [20] analysed the essential oil from *Zanthoxylum clava*-*herculis* and found the content of eucalyptol ranged from 16 to 43%. Similar research was also carried out on the essential oil from the fruits of *Zanthoxylum bungeanum* [21]; *Zanthoxylum rhetsoides* and *Zanthoxylum myriacanthum* [22], the content of eucalyptol is 16.0% 15.7% and 18%, respectively. β-Caryophyllene, is also a common constituent in many *Zanthoxylum* essential oils. It reported to be found in the essential oils of *Zanthoxylum newbouldia* leaves (36%) [23]; *Zanthoxylum syncarpum* (9.2–9.3%) [24]; *Zanthoxylum acanthopodium* leaves (3.0%) [25]; *Zanthoxylum rubescens* leaf (22.1%) [26]; *Zanthoxylum setulosum* (13.7%) [27]; *Zanthoxylum ekmanii* (11.5%) [28]; *Zanthoxylum procerum* leaves (7.0%) [29]. The results indicate that the *Z. acanthopodium* essential oil shares some similar main constituents with other species of *Zanthoxylum*.

### Larvicidal assays

Tables 2, 3 displays the results on percent mortality of larvae of *An. sinensis* and *An. anthropophagus* with increase in essential oil and test compounds concentration. The mortality of *An. sinensis* was 17, 54, 68, 83, 94 and 100% when the oil at concentration of 25.0, 50.0, 100.0, 125, and 150 mg/L. The mortality of *An. anthropophagus* was 36, 62, 78, 90, 97 and 100% when the oil at concentration of 25.0, 50.0, 100.0, 125, and 150 mg/L. The LC_50_ and LC_90_ values against *An. sinensis* larvae were 49.02 and 125.18 mg/L and for *An. anthropophagus* were 36.00 and 101.49 mg/L, respectively. Between two compounds tested for 24 h, estragole exhibited a stronger mosquito larvicidal activity than the oil with LC_50_ 41.67, 38.56 mg/L and LC_90_ 107.89, 95.90 mg/L for *An. sinensis* and *An. anthropophagus.* Eucalyptol had less LC_50_ values (45.49 and 42.41 mg/L) and LC_90_ values (124.95 and 111.45 mg/L).

**Table 2:** Larvicidal activity of *Zanthoxylum acanthopodium* essential oil, estragole and eucalyptol against fourth instar *Anopheles sinensis* larvae

Compounds	Concentration (mg/L)	24 h mortality (%)	LC_50_ (mg/L) (95% CI)	LC_90_ (mg/L) (95% CI)	Slope ± S.E.	Chisquare (df)
Essential oil	25	17	49.02 (44.15–49.41)	125.18 (105.90–141.74)	3.29 ± 0.28	2.835 (3)^a^
	50	54				
	75	68				
	100	83				
	125	94				
	150	100				
Estragole	25	28	41.67 (29.48–52.07)	107.89 (82.90–178.83)	3.10 ± 0.28	5.994 (3)^a^
	50	57				
	75	72				
	100	89				
	125	97				
	150	100				
Eucalyptol	25	26	45.49 (33.24–56.33)	124.95 (94.78–213.84)	2.92 ± 0.27	5.329 (3)^a^
	50	51				
	75	69				
	100	83				
	125	95				
	150	100				
DMSO		1 ± 0.45				

Each datum represents the mean of five replicates, each set up with 20 individuals (n = 100)

LC values are considered significantly different when 95% CI fail to overlap

*95% CI* confidence interval at 95% confidence level

^a^The significance level is more than 0.05. A heterogeneity factor is used in the calculation of confidence limits

**Table 3:** Larvicidal activity of *Zanthoxylum acanthopodium* essential oil, estragole and eucalyptol against fourth instar *Anopheles anthropophagus* larvae

Compounds	Concentration (mg/L)	24 h mortality (%)	LC_50_ (mg/L) (95% CI)	LC_90_ (mg/L) (95% CI)	Slope ± S.E.	Chisquare (df)
Essential oil	25	36	36.00 (30.90–40.61)	101.49 (88.49–121.70)	2.85 ± 0.27	3.885^a^
	50	62				
	75	78				
	100	90				
	125	97				
	150	100				
Estragole	25	31	38.56 (26.73–48.40)	95.90 (74.43–153.82)	3.24 ± 0.29	6.248 (3)^a^
	50	60				
	75	78				
	100	91				
	125	99				
	150	100				
Eucalyptol	25	28	42.41 (37.49–47.01)	111.45 (97.62–132.64)	3.05 ± 0.27	4.749 (3)^a^
	50	54				
	75	73				
	100	88				
	125	96				
	150	100				
DMSO		2 ± 0.55				

LC values are considered significantly different when 95% CL fail to overlap

*95% CI* confidence interval at 95% confidence level

^a^The significance level is more than 0.05, a heterogeneity factor is used in the calculation of confidence limits

Mosquitoes, including *An. sinensis* and *An. anthropophagus* used in these experiments, act as vectors for many disease-causing viruses and cause serious health problems worldwide in both humans and animals. Although *Zanthoxylum* is a known potential source of anthelmintic agents for traditional Chinese medicine, only a few studies have assessed the anti-mosquito activity of *Zanthoxylum* species. Trongtokit et al. [30, 31] showed that the essential oil from *Zanthoxylum limonella* had repellent effect against *Aedes aegypti*, *Culex quinquefasciatus*, and *Anopheles dirus*. Effects of *Zanthoxylum armatum* essential oil against *Aedes aegypti* (LC_50_ = 54 ppm), *Anopheles stephensi* (LC_50_ = 58 ppm) and *Culex quinquefasciatus* (LC_50_ = 49 ppm) was analysed by Tiwary et al. [32]. The essential oil from leaves of *Zanthoxylum articulatum* was examined with respect to its larvicidal properties against the larvae of *Aedes aegypti* and showed that LC_50_ was 77.62 ppm [33]. Essential oil of *Zanthoxylum piperitum* had insecticidal effect against *Aedes gardnerii*, *Anopheles barbirostris*, *Armigeres subalbatus*, *Culex tritaeniorhynchus*, *Culex gelidus*, *Culex vishnui* group, and *Mansonia uniformis* [34]. The related activity of *Zanthoxylum beecheyanum* was also reported, 24 h LC_50_ was 6.895 mg/mL against *Culex pipiens* adults and 119.020 mg/mL against 4 instar larvae [35].

When comes to the larvicidal mechanism of the *Zanthoxylum* essential oil, it can be attributed to some compounds in the oil that can lead to alteration in the membrane structure of larval cells [36, 37], especially estragole, eucalyptol, β-caryophyllene and limonene, whose separated insecticidal effect have been revealed in a series of reports. Zhang et al. [38] showed estragole had strong contact toxicity against *Lasioderma serricorne* adults with 24 h LD_50_ value of 15.58 mg/adult, and in the fumigant toxicity test, the 24 h LD_50_ was 5.18 mg/L air. When against *Maize weevils*, 24 h LC_50_ values of estragole was 14.10 ppm [39]. Kimbaris et al. [40] showed that 24 h LC_50_ of eucalyptol was inactive at concentrations even as high as 100 mg/L against early fourth instar mosquito larvae of *Culex pipiens*. Against the 3rd instars of the *Culex pipiens* 24 h LC_50_ values of eucalyptol was 91.45 mg/L [41]. The current study proved that estragole and eucalyptol played important role as insecticidal compounds in *Zanthoxylum acanthopodium* essential oil against two anopheline mosquito species. Yang et al. [42] showed that eugenol was the most significant compounds of *Clove Bud* oils with reference to repellent activity against the bean bugs *Riptortus clavatus*. You et al. [43] showed that β-caryophyllene, exhibited strong insecticidal and repellent activities against *Lasioderma serricorne*. Wang et al. [39] showed the limonene LC_50_ = 6.21 mg/L for *Tribolium castaneum* and 14.07 mg/L air *for L. serricorne*.

To sum up, a series of investigations for chemical compounds from natural products have revealed that some essential oil with adequate active ingredients is essential to the development of new insecticidal drugs, especially in view of the vast worldwide flora. There is also some evidence indicating that essential oils often prove to be more effective than their components, indicating synergy [44].

## Conclusion

In total, 63 main compounds (99.32%) were found in *Zanthoxylum acanthopodium* essential oil, including estragole (15.46%), eucalyptol (10.94%), β-caryophyllene (5.52%), *cis*-linalool oxide (3.76%), *cis*-limonene oxide (3.06%). Both whole essential oil and its main compounds (estragole and eucalyptol) showed significant larvicidal activity. The results revealed that *Zanthoxylum acanthopodium* essential oil could be further developed as a potential agent to control the larvae of malaria mosquito.
